# Review on Fabrication Technologies for Optical Mold Inserts

**DOI:** 10.3390/mi10040233

**Published:** 2019-04-03

**Authors:** Marcel Roeder, Thomas Guenther, André Zimmermann

**Affiliations:** 1 Hahn-Schickard, Allmandring 9b, 70569 Stuttgart, Germany; zimmermann@ifm.uni-stuttgart.de; 2 Institute for Micro Integration (IFM), University of Stuttgart, Allmandring 9 b, 70569 Stuttgart, Germany; thomas.guenther@ifm.uni-stuttgart.de

**Keywords:** optical mold inserts, micro machining, micro structuring, ultra-precision machining, mold fabrication

## Abstract

Polymer optics have gained increasing importance in recent years. With advancing requirements for the optical components, the fabrication process remains a challenge. In particular, the fabrication of the mold inserts for the replication process is crucial for obtaining high-quality optical components. This review focuses on fabrication technologies for optical mold inserts. Thereby, two main types of technologies can be distinguished: fabrication methods to create mold inserts with optical surface quality and methods to create optical microstructures. Since optical mold inserts usually require outstanding form accuracies and surface qualities, a focus is placed on these factors. This review aims to give an overview of available methods as well as support the selection process when a fabrication technology is needed for a defined application. Furthermore, references are given to detailed descriptions of each technology if a deeper understanding of the processes is required.

## 1. Introduction

Polymer optics have gained increasing importance in recent years. They compete with traditional glass lenses in various fields of applications. One of the most critical points in the fabrication of polymer optical components is the mold insert required for injection molding or injection compression molding, respectively. There is a broad range of technologies which can be used to produce optical mold inserts and/or micro-structuring techniques. Which methods should be employed depends mainly on the application. This review aims to support decision making when selecting the most suitable fabrication technology by providing an overview of available technologies. The scope of this review is to describe the technologies, their advantages and limitations, and possible applications. Since the review focuses on optical mold inserts, special attention is given to achievable surface quality, accuracy and, in the case of micro-structuring techniques, the minimal structure size.

The market of polymer optics is growing rapidly, finding its way into more and more sophisticated applications [1]. Technological advantages in the fabrication process of polymer optics enable fast replication of optical elements with a wide range of geometries as well as micro-structures. This is a major advantage compared to glass optics, allowing for more freedom in optical design. Free-form optics are just one example of optical element that can be produced at a significantly lower price than traditional glass lenses. Therefore a wide range of applications emerges with increasing opportunities for the optical design. Applications range from illumination [2] and imaging [3] to automotives [4].

Another upcoming trend in polymer optics are micro-structured components. The combination of lenses with micro-structured features can be used to increase their performance, reduce the weight of optical systems, correct aberrations, and shape beams. Examples of micro-structures used in polymer optics are micro-lens arrays [5], diffractive optical elements [6], Fresnel lenses [7], prism arrays [8] and blazed structures [9]. Examples of applications of micro-structured optical components are concentration structures for solar panels using micro-lens arrays [10] or Fresnel lenses [11], beam shaping and homogenization [12,13], measurement systems [14] and sensors [15,16].

One of the main advantages of polymer optics is their fast and low-cost replication by means of hot embossing [17] or injection (compression) molding [18]. Furthermore, mounting and alignment features can be integrated into the optical components, which eliminates the need for additional holding components and assembly steps [19]. Roll-to-roll processes enable the fast replication of large areas with an accuracy even appropriate for micro-structured features [20,21]. While this opens further technological possibilities, this paper does not focus on replication technologies, but on the fabrication of mold inserts for the replication. A comprehensive overview on injection molding of polymer optics is given by Bäumer [22]. Furthermore, methods for the replication of micro- and nano-structured surface geometries are summarized by Hansen et al. [23].

The most important material properties for polymer optics are the refractive index and the Abbe number [1]. Comparing polymer optics to glass optics, the refractive index is a limiting factor since no materials with high refractive indices are available. The most commonly used materials for injection molded optics are acrylic (PMMA), polycarbonate (PC), cyclic olefin copolymer (COC) and cyclic olefin polymer (COP) [24], which provide good technical properties regarding internal stresses, reduced water absorption, optimized resistance against environmental influences, and many more. In combination with microstructured features, their optical properties can be enhanced to overcome the limitations concerning refractive index.

In the following section, technologies for the fabrication of optical mold inserts are described. The technologies are divided in form-giving methods and micro-structuring techniques. First, form-giving machining technologies for optical mold inserts are described, where ultra-precision machining presents a special case as it presents a combination of a form-giving and micro-structuring technology. The methods are investigated regarding their achievable surface quality and accuracy. Subsequently, micro-structuring methods are described, focusing on the achievable structure size. A summary is provided in the last section of the paper including a guide for aiding decision making when choosing the right technology for the required application.

## 2. Fabrication Methods

**Figure 1:**
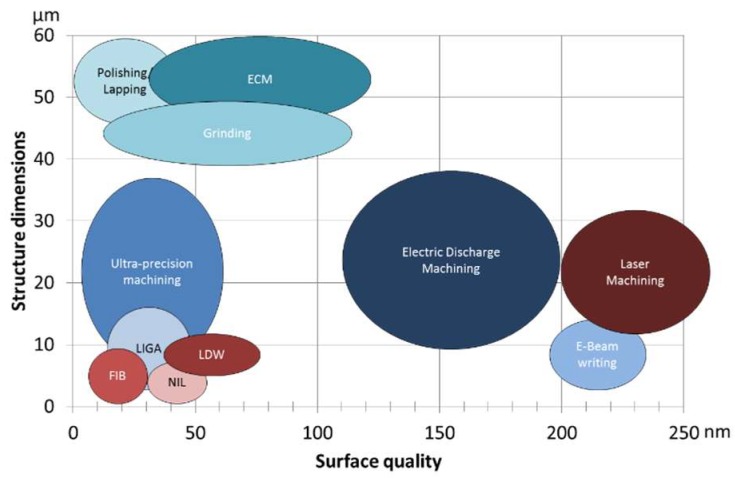
Structural dimensions and achievable surface quality of fabrication technologies for optical mold inserts.

Throughout this section, various fabrication methods are described. Figure 1 provides an overview of the methods and their achievable surface quality and structure dimensions. More details of each technology are described below.

## 3. Form-Giving Technologies

### 3.1. Ultra-Precision Machining (UPM)

Ultra-precision machining (UPM) was first introduced in the 1960′s by Bryan from the Lawrence Livermore National Laboratory [25]. It is the most common method for the fabrication of optical mold inserts. Ultra-precision machines achieve a positioning accuracy in the nanometer range [26], which leads to outstanding surface quality and form accuracy. The surface roughness of diamond machined parts is usually smaller than Ra < 10 nm. Hence, post-processing of components to achieve mirror finished surfaces is not required.

To obtain high-quality parts, the machine components have to be pushed to their limits. Diamond machining systems use a granite block as a foundation. High-precision positioning systems, high-speed spindles, and accurate fixture and handling equipment are needed in these systems [27]. The current state of the art in spindle technology was reviewed by Abele et al. [28]. Air bearing spindles and oil hydrostatic bearings are used for accurate movement of the tools and parts. Position control is assured by glass scales with a resolution of less than 1 nm. Furthermore, vibration suppression and temperature control is very important. Temperature should be kept constant in a range of ±0.1 K or less. When machining at the microscale, mechanics change significantly. Effects which have little to no influence at macroscale become dominant when the chip size decreases. The achievable surface roughness of diamond-machined parts is influenced by a multitude of factors like cutting conditions [29], tool vibration [30,31,32], material properties [33,34,35,36,37,38,39] and spindle vibration [40]. These factors can be separated into process factors and material factors [29]. The understanding of the effects and their impact on surface roughness is most important to improve part quality and support further development of the technology. Cheung and Lee [29,32,41,42,43] investigated the cutting dynamics and surface generation in ultra-precision machining, mainly using diamond turning as the cutting technology.

The achievable part quality and accuracy very much depends on the quality of the diamond tool. Monocrystalline diamonds are used to form the cutting tip of the tool because of their outstanding hardness and the ability to create very sharp edges with less than 50 nm edge roundness [44]. Hence, the surface finish does not depend on the cutting speed [45]. Ultra-precision machining can also be used as a micro-structuring technique. The achievable structure size is thereby limited by the nose radius of the available diamond tools to about 5 µm [46].

Diamond machining is limited to non-ferrous materials. Due to this fact, nickel-phosphorus (NiP) coatings became the industry standard as the material to machine with diamond tools for optical mold inserts. NiP can be diamond machined with negligible tool wear. The coatings are deposited onto steel molds by electroless or galvanic plating processes. The preparation of the mold inserts before the diamond machining requires three steps. First, a steel insert is fabricated using traditional milling or turning processes to fabricate the geometry roughly. Afterwards, the nickel phosphorous coating is deposited and another rough machining process is needed to remove surplus NiP since the diamond machining process only removes a couple of micrometers of the material.

**Figure 2:**
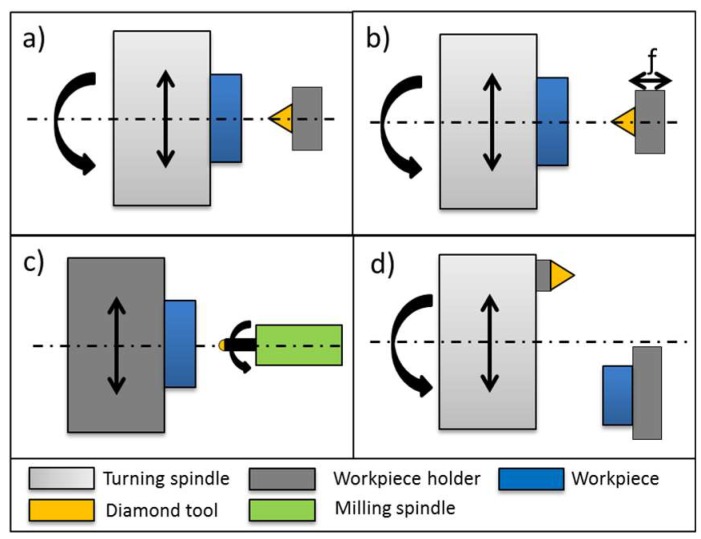
Ultra-precision machining (UPM) methods. (**a**) Diamond turning; (**b**) Slow-tool-servo/fast-tool-servo (**c**) Diamond milling; (**d**) Fly cutting.

The necessity of the coating process makes the fabrication of optical mold inserts by means of diamond machining costly and time consuming. Therefore, efforts are made to machine tool steel inserts directly with optical surface quality. Different methods of UPM as well as methods to machine steel-based materials will be discussed in subsequent sections. Machining configurations of UPM are shown in Figure 2 and will be explained in the following sections.

#### 3.1.1. Diamond Turning

Early developments of ultra-precision machining where driven by the demand for large size lenses with high-quality surfaces, mainly produced by diamond turning. Diamond turning is used to fabricate rotationally symmetrical components with high accuracy and a surface roughness Ra < 10 nm [47]. Ikawa et al. [48] reported the minimum chip thickness achievable in diamond turning is 1 nm in an experimental setup. Due to the machine configuration, possible part geometries are limited. Diamond turning is a standard process to fabricate optical mold inserts for spherical and aspherical lenses. Riedl [49] and Blough et al. [50] reported the fabrication of a diffractive optical element by means of diamond turning. When diamond turning is used to create micro-structures, the structure size is limited by the available diamond tools to about 5 µm [46].

**Figure 3:**
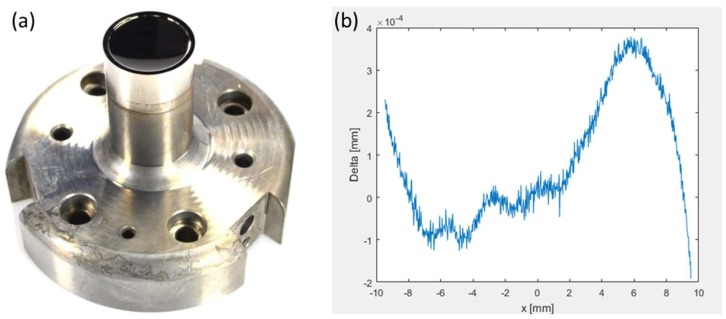
(**a**) Diamond turned mold insert, (**b**) form deviation of the optical aspheric surface with P-V < 1 µm (Peak to Valley).

Beside cutting conditions and machine properties the achievable surface quality of diamond-turned parts very much depends on process and material factors. The influence of process factors can be reduced or even eliminated by optimizing the operation settings. The main factors influencing the part quality are the spindle speed, tool tip radius and feed rate. Cheung and Lee [29] reported that high spindle speed, large tool tip radius and slow feed rate generally improve surface roughness. Most machines are able to rotate at a maximum speed of about 5000–6000 RPM (rounds per minute). To achieve high form accuracy the vertical and horizontal positon of the tool tip is very sensitive. Deviations lead to a residual cone in the center or an error in the surface radius. An example of a diamond-turned optical mold insert and the resulting form accuracy are shown in Figure 3.

##### Slow Tool Servo

Due to the high demand for non-symmetrical optics, slow tool servo (STS) was developed. Additional to the classical diamond turning setup, the z-axis oscillates during the process. Thus, the contact of the tool tip is intermittent. The slow tool servo is able to oscillate in the area of about 25 mm. Furthermore, the c-axis has to be controlled to coordinate the tool and workpiece position [51]. A slow tool servo is able to produce very accurate asymmetrical parts without any additional machine equipment. The spindle speed in these processes is typically lower compared to regular diamond turning with a rotation speed of maximum 2000 RPM. For a good result, the position accuracy and the coordination of the axis are very important [52]. Similar to the diamond turning process, an accurate tool tip position is very sensitive to the fabrication result. The machining time is long compared to fast tool servo (FTS) or diamond milling due to the fact that the z-axis is massive and can only achieve limited speed [53]. Using the STS method, surface roughness better than 10 nm is achievable [54]. The technology can also be used to fabricate micro-structured components. The structure sizes are limited by the available diamond tools and their nose radius to about 5 µm. STS is used to fabricate different optical components or optical mold inserts like micro lens arrays [55], prism arrays [56], diffractive optical elements [54], off-axis aspheric surfaces [57], freeform optical surfaces [58,59] and molds for compound eye lenses [60].

##### Fast Tool Servo

**Figure 4:**
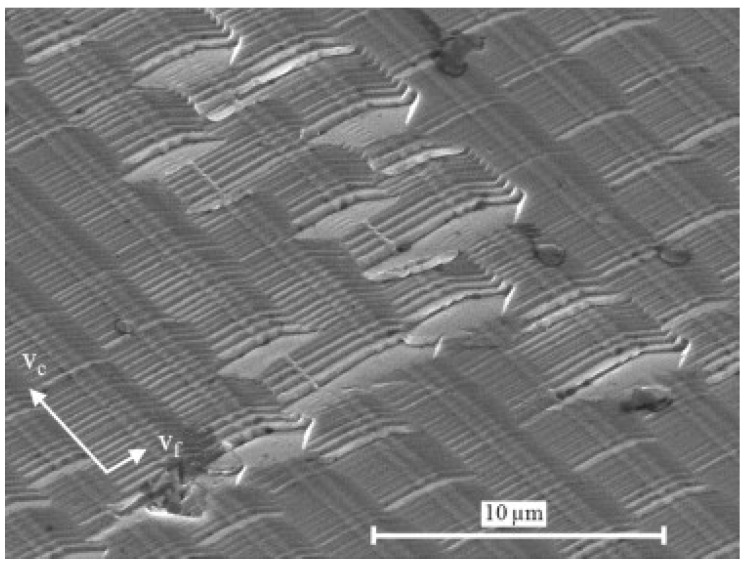
Scanning electron microscope (SEM) image of a diffractive surface generated by fast tool servo (FTS) in nickel silver. Reproduced with permission from [62].

The machine setup for a fast tool servo is very similar to the STS configuration with a rotating workpiece and an oscillating tool. In contrast to STS, for the FTS machining an additional actuator for the tool is necessary, which oscillates the tool tip. The fast tool servo allows an accurate positioning of the tool but is limited by the stroke which is significantly smaller compared to STS technology [57]. Strokes usually are in the range of a few micrometers to a few hundred micrometers. Some FTS systems are optimized for either very short strokes <1 µm or long strokes up to 1 mm [61,62]. Different FTS technologies are reviewed by Trumper and Lu [63]. In a FTS setup, the spindle has an encoder which feeds the positon to the FTS, but does not put the spindle in position control [57]. FTS is commonly used for the fabrication of diamond-turned surfaces with structures like micro prisms, lens arrays, torics and off-axis sags with small sags [64]. FTS is also a suitable technology for the fabrication of diffractive optical elements as shown in Figure 4. Brinksmeier et al. reported the fabrication of a diffractive optical element with submicron structure size [62]. Resulting surfaces with roughness Ra <10 nm are possible [65].

#### 3.1.2. Diamond Milling

In the diamond-milling process a diamond ball end mill tool with one cutting edge is used. The tool rotates at very high speed and thereby removes chips in the micrometer range. In contrast to the diamond turning process, the cutting speed is not accomplished by the rotation of the workpiece but by a fast rotation of the milling tool. Furthermore at least three controlled axes are needed. For the tool rotation, usually an air bearing spindle with very low error motion in the nanometer range is used. The rotational speed can be up to 100,000 RPM and more. For the positioning of the tool, three or more axes are used. Compared to diamond turning, the milling process is significantly slower but offers great freedom in the design. For the diamond-milling process the quality of the milling tool is very important, Therefore, the diamond has to be centered perfectly on a cylindrical shank [53,66].

Compared to the diamond-turning process, milling is much slower due to a small material removal rate. Hence, diamond milling is mainly used for non-smooth surfaces where turning processes are not applicable.

**Figure 5:**
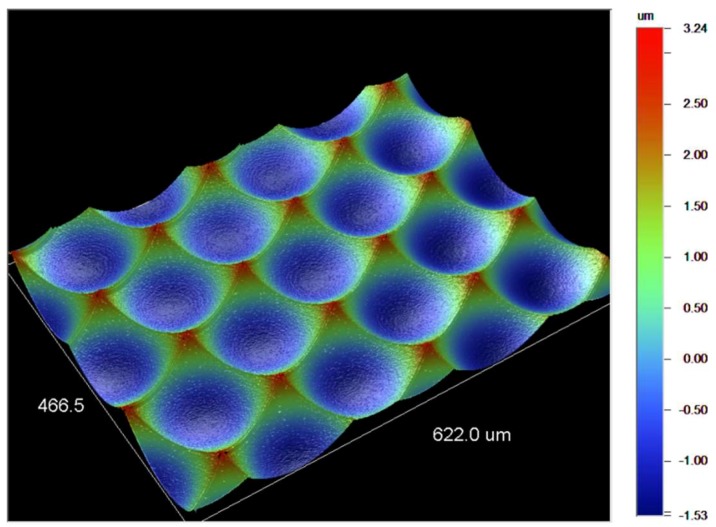
Micro lens array fabricated by means of diamond milling measured by white light interferometry (WLI).

Diamond milling is often used for the fabrication of micro lens arrays [66], as shown in Figure 5 and free-form surfaces [64]. Milling experiments conducted in nickel-phosphor for the fabrication of a micro lens array resulted in surface quality Ra <10 nm.

#### 3.1.3. Fly Cutting

**Figure 6:**
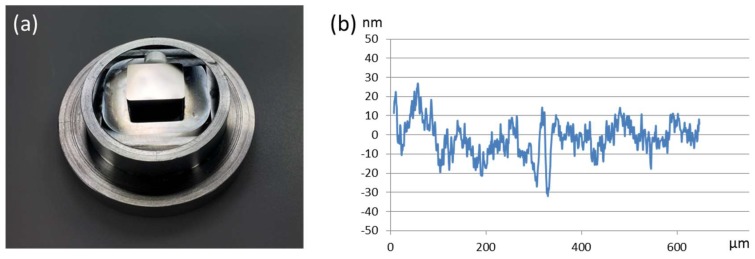
(**a**) Optical flat surface on a mold insert fabricated by fly cutting; (**b**) Resulting surface roughness of Ra = 8 nm measured by WLI.

Fly cutting processes use a rotating tool whereby the diamond is placed off-axis on the tool. Therefore, the diamond tool is not permanently in contact with the material. Fly cutting can be used to create planar surfaces efficiently with optical surface quality, also on large areas. Furthermore, fly cutting is a suitable method to create microstructures and free form optics. Example for micro-structures are Fresnel lenses [67], micro pyramid arrays [68] and diffractive gratings [69]. Brecher et al. used the fly-cutting process for the fabrication of a complex free form mold [70]. The achievable form accuracy is in the sub-micrometer range and the resulting surface quality in the nanometer range [71]. The fly-cutting technology can also be used to create large plane surfaces quickly with a surface roughness Ra <10 nm. Figure 6 shows a flat optical surface fabricated by fly cutting with a surface roughness of Ra = 8 nm.

#### 3.1.4. UPM of Steel

As already mentioned, diamond machining is usually limited to polymers and non-ferrous metals. Since hardened steels are the most popular engineering material, a lot of research has been conducted to achieve the machinability of ferrous materials with diamond tools in order to use the benefits of diamonds as a cutting material. A good review on diamond cutting of ferrous metals is given by Li et al. [72]. The review not only focuses on different methods for diamond cutting of ferrous materials but also on the wear mechanisms that are at work during the cutting process. The following wear mechanisms can be distinguished [73]:

- Adhesion and formation of a built-up edge;
- Abrasion, microchipping, fracture and fatigue;
- Tribothermal wear;
- Tribochemical wear.

The main effects for tool wear are chemical mechanisms. Paul et al. further divided the chemical mechanisms into diffusion, oxidation, graphitization and carbide-formation and linked the wear to the presence of unpaired d-electrons in the metal [74]. Metals with unpaired d-electrons lead to strong wear on the diamond tool while tool wear is negligible when machining metals without unpaired d-electrons. Diffusion of carbon atoms into the ferrous material takes place after the diamond tool graphitizes on the surface [75]. Molecular dynamics simulation supported these investigations and helped to gain a deeper understanding of the mechanisms on the atomic and nanometer scale [76,77].

To avoid severe tool wear a lot of research has been conducted to create methods which reduce the tool wear of diamond when cutting ferrous materials. Proposed methods are:

- Using ultrasonic vibration cutting;
- Optimizing cutting conditions;
- Modifying the cutting tool;
- Using binderless cubic boron nitride (cBN) tools.

Ultrasonic vibration cutting is the most promising method for the machining of ferrous materials using a diamond tool. Moriwaki and Shamoto were the first to propose a vibrating cutting tool to reduce tool wear [78]. The cutting tool is elliptically vibrating and, therefore, significantly reducing friction force and contact time of the diamond with the substrate [79]. Because of the elliptical movement of the diamond tool the technology is often referred to as elliptical vibration cutting. Different variations of the technology and applications are reviewed by Zhang et al. in [80]. Ultrasonic vibration cutting can be used in a turning process or with a moving x/y/z stage. The technology is not just very useful for the machining of ferrous materials with a diamond tool but also enables the micro-structuring of surfaces with a broad range of structures like V-grooves and pyramids as well as free forms [81,82]. At the same time, optical surface quality with Ra <10 nm is achievable [83].

##### Optimized Cutting Conditions

Another approach to reduce diamond wear is to modify the cutting conditions. Research teams tried different cutting conditions like reduced temperature and machining under a gaseous environment. Evans and Bryan performed diamond turning under cryogenic conditions and reported significantly reduced tool wear and achieved a surface roughness better than 25 nm [73]. Due to the low temperature during the cutting process, chemical reactions are slowed down significantly which leads to reduced tool wear. Casstevens examined diamond tool wear when machining steel under a carbon-rich atmosphere and found that tool wear under a methane environment reduces tool wear significantly and allows to achieve optical surface finish with Ra <12.5 nm [84]. However, other research groups report contrary results. Machining under inert gas environment like argon and nitrogen does not reduce the tool wear compared to air, as reported by Brinksmeier and Gläbe [85].

##### Tool Modification

Another method to increase wear resistance of diamond tools are protective coatings. Thus, a direct contact between the diamond and the ferrous material can be prevented. Klocke et al. discuss different coated tools for metal cutting, but did not focus on diamond tools [86]. Brinksmeier and Gläbe used TiN and TiC coatings on diamond tools to reduce tool wear and found, that flank wear is reduced as long as the protective coating is still in place but the layers are removed abrasively during the cutting process [85]. This leads to the conclusion, that coatings can prevent chemical wear but suffer from abrasive wear.

##### Binderless Cubic Boron Nitride (cBN)

**Figure 7:**
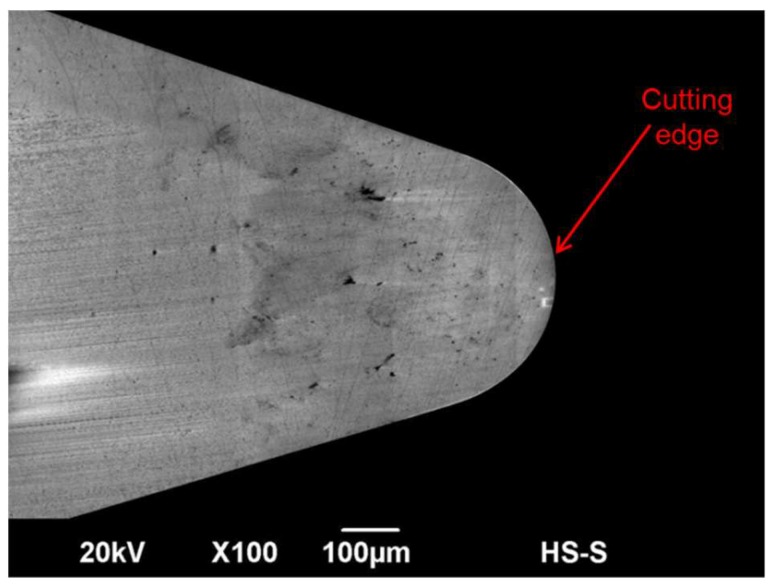
Scanning electron microscope (SEM) image of binderless cubic boron nitride (cBN) cutting tool.

One of the most promising methods to obtain optical surfaces in ferrous materials is the use of binderless cubic boron nitride tools (binderless cBN). cBN shows a very good heat and chemical resistance and has the second-highest hardness after diamond [87]. Thereby, very small grain sizes are needed to achieve low surface roughness. Binderless cBN tools show a strong wear resistance against adhesion, abrasion and attrition [88]. Uhlmann et al. reported a surface roughness of Ra <10 nm using a binderless cBN tool in a turning process of stainless steel with a hardness of H = 52 HRC [89]. While cBN tools are already widely used as a cutting material the availability of binderless cBN tools is still very limited. Regular cBN tools are usually sintered with binder metals, which are mainly responsible for reduced wear resistance [87]. A scanning electron microscope (SEM) image of a binderless cBN tool is shown in Figure 7.

### 3.2. Electric Discharge Machining (EDM)

Electric discharge machining (EDM) is a thermo-electric machining process in which a series of electrical discharges between the tool electrode and the workpiece removes material [90]. It is important to mention that the discharge sparks produced in the gap between tool electrode and workpiece remove material on both parts by melting and evaporation. The whole process is performed in a dielectric medium. The EDM process is limited to conductive or semi-conductive materials.

There are a number of different process variants for the EDM technology. A good overview is given by Uhlman et al. [91]. Using EDM for the fabrication of optical mold inserts is a relatively new approach. Takino and Hosaka proposed the use of EDM for the fabrication of a micro lens mold insert [92]. Thereby a ball-type electrode is used for shaping the mold insert made of stainless steel. In [93] they propose to use a nine-ball electrode, so that multiple lenses can be fabricated simultaneously. The form accuracy was reported to be 10 µm with a surface roughness of 0.85 µm. For optical applications this is not sufficient and post-processes like grinding, cutting or polishing are necessary to obtain smooth and accurate optical surfaces. But with further optimization of the EDM process the surface quality could be improved. Other publications report an achievable surface roughness of >0.3 µm using the EDM technology but in those cases the aim was not to produce optical mold inserts [91,94].

**Figure 8:**
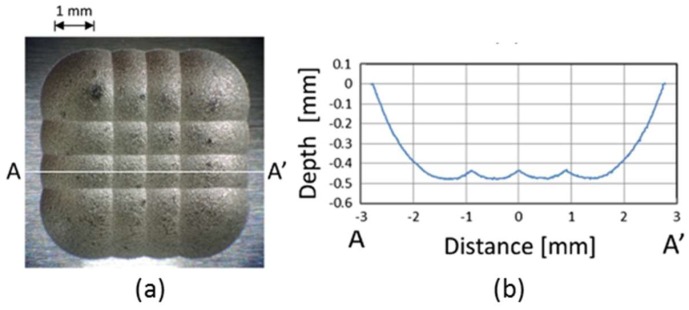
Micro lens array mold insert fabricated by electric discharge machining (EDM). (**a**) Mold surface observed with optical microscope, (**b**) cross section of the surface along line AA’. Reproduced with permission from [92].

Micro-EDM can also be used to fabricate micro-structures. The technology is especially powerful, when micro-structures with high aspect ratios are needed [95]. Structure sizes down to 3 µm are possible and aspect ratio can be as high as 100 [96]. An extensive review about issues with the micro-EDM process is given by Pham et al. [97]. An example of an optical mold insert fabricated by EDM technology is shown in Figure 8.

### 3.3. Electrochemical Machining (ECM)

Electrochemical machining (ECM) uses the anodic dissolution of metals during an electrolysis process for the material removal [98]. Similar to the EDM process, the machining shape is defined by the shape of the electrode and the workpiece has to be conductive. The resulting surface is very smooth and can be used for the smoothing of micro-metallic products [99]. Compared to traditional machining technologies, ECM has several advantages like high material removal rate, applicability regardless of the material hardness, no tool wear and smooth surfaces [98]. The technology can be used for post-processing a regularly machined workpiece. The technique is then referred as electrochemical polishing. The quality of the resulting surface is depending severely on the type of the electrolyte and the workpiece material and can reach optical quality [100]. Using a modified ECM process, Kurita and Hattori achieved a surface roughness of 0.06 µm [101]. But at this point, optical applications remain very rare. The process is more common for the elimination of burr in molds [102].

To decrease the removal rate to a minimum, short pules and a low current are required [99]. When a pulsed current is used instead of DC current, the technology is referred as PECM. Thus, the machining accuracy and process stability can be improved [90]. De Silva et al. used this process on chrome steel and achieved a surface roughness of 0.03 µm with a form accuracy better than 5 µm [103]. The main factors affecting the process are the electrolyte, the electrolyte flow conditions in the inter-electrode gap and the gap size [90].

When ECM is combined with the STM (scanning tunneling microscope) technology, microgrooves with submicron width can be achieved [99].

### 3.4. Grinding

Grinding is commonly used for the fabrication of optical molds. Therefore a fine grinding process is used to achieve high form accuracy. Since the achievable roughness during the grinding process is not sufficient for optical applications a post treatment process like polishing is mandatory. Recent developments aim to improve surface quality to overcome the necessity of a subsequent polishing step in the future. Polishing will be discussed in this review in a following section. Using a grinding process, surface roughness in the nanometer range can be achieved [104,105], but the results depend strongly on the tool wear, workpiece material and process parameters. In this paper, focus is placed on ultra-precision grinding since for optical applications high accuracy and low surface roughness are required. An extensive review of ultra-precision grinding has been provided by Brinksmeier [106]. In contrast to polishing and lapping, grinding uses fixed abrasives which are in interrupted contact with the workpiece. A main driving factor for grinding is the possibility to machine brittle and hard materials like ceramics, glass, carbides, glasses, hardened steels and semiconductor materials which can with difficulty be machined with ultra-precision machining [106]. Grinding processes can produce very accurate surfaces but need long machining times [57].

For optical applications, grained diamond wheels or cBN wheels are often used to achieve good form accuracy and surface roughness with Ra < 10 nm [107]. An important factor to produce high quality surfaces by grinding is to ensure a stable condition of the grinding wheel. A suitable method is the electrolytic in-process dressing (ELID) process, first proposed by Nakagawa and Ohmori [108].This in-process dressing prevents high wheel wear resulting in stable grinding conditions.

The main applications in the optical field are the manufacturing of spherical glass lenses or molds for glass injection molding [104].

## 4. Micro-Structuring Technologies

### 4.1. Lithographie, Galvanik and Abformung (LIGA)

The LIGA technology was developed at Karlsruhe Nuclear Research Center by Becker and Ehrfeld in the 1980s [109]. LIGA stands for the three German words Lithographie, Galvanik and Abformung. The LIGA technology is broadly used for the fabrication of injection molding tools [110,111]. For parts with high aspect ratio structures, the technology has particular advantages compared to other fabrication technologies [112]. Micro-structures <1 µm can be produced using this technology [113]. The LIGA technology describes a process chain of three consecutive processes. The first step is a lithography process for the structuring of a substrate. Afterwards, a nickel electroplating process is performed to create a mold using the structured substrate as a master. A detailed description of this process can be found in [114]. In the final step, parts can be produced using injection molding or hot embossing. The process chain is described in detail in [109].

**Figure 9:**
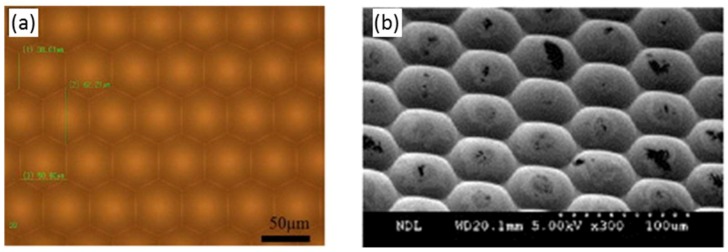
(**a**) Mold insert with a micro lens array fabricated using the Lithographie, Galvanik and Abformung (LIGA) process and (**b**) replicated micro lenses in a polymer film. Reproduced with permission from [123].

A main application of the LIGA process in the optical field is the fabrication of DOE (diffractive optical elements) [115,116,117,118,119]. However, lithography for diffractive optical elements is expensive and time-consuming when the DOE level increases to over 4 [30]. A modified LIGA process can also be used for the fabrication of micro lens arrays with a resulting surface roughness of 1 nm [120], which is shown in Figure 9. Further optical components that can be fabricated with the LIGA technology are micro-prisms [8], micro-mirrors [121] and waveguides [122].

### 4.2. Nanoimprint Lithography (NIL)

**Figure 10:**
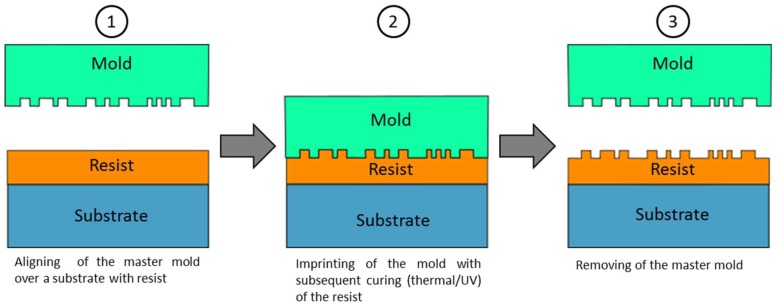
Nanoimprint lithography (NIL) process chain.

Nanoimprint lithography (NIL) is a lithographic technique that allows high-throughput patterning of polymer nanostructures. The structures can be produced with a high accuracy at low costs. The process was first described by Chou et al. in 1995 [124]. NIL describes a process consisting of three main steps. First, a master mold is fabricated using a micro structuring technology like electron-beam lithography or interference lithography. In a second step the master structure is duplicated into a mold. The mold can be hard, soft or a hybrid of those [125]. The final step is the imprint process. Thereby the mold structure is imprinted into a resist on a substrate which leads to a transfer of the microstructures. Afterwards, the resist has to be cured. There are two variations of the NIL process. Thermal NIL uses heating of the resist above the glass transition temperature during the imprint step with subsequent cooling to room temperature. UV-NIL uses ultraviolet light to cure the resist. Therefore, the mold hast to be transparent. An extensive review on NIL focusing on different process variations, required material properties and nanostructure replication is given by Guo [126]. A schematic of the nanoimprint process is shown in Figure 10.

Using the NIL technology nanostructures with features sizes <10 nm can be produced and replicated [127]. Therefore, NIL is often used for photonics applications since the optical surface properties of substrates can be controlled precisely. Applications are holograms, diffractive structures, anti-reflective structures, micro-lens arrays and roll-to-roll applications. Since nanoimprint lithography is a method for patterning nanostructures, surface roughness is not a focus of the research.

### 4.3. Laser Direct Writing

In contrast to laser machining, laser direct writing (LDW) uses a laser beam for the structuring of a photoresist, comparable to a lithography process used in semiconductor manufacturing. A thin film of photoresist is deposited on a substrate. Afterwards, the LDW process is used for the structuring of the photoresist. Therefore, the substrate is scanned by a focused laser beam whereby the laser intensity is synchronously modulated [114]. In the final step the photoresist has to be developed. The LDW process is described in detail in [128].

LDW allows the fabrication of binary and continuous structures [114]. LDW is very often used for the fabrication of Fresnel or diffractive structures, mostly on planar substrates [129] where continuous structuring is beneficial for the optical performance. Compared to a lithographic approach, LDW avoids the need for submicron alignment for consecutive exposure steps.

**Figure 11:**
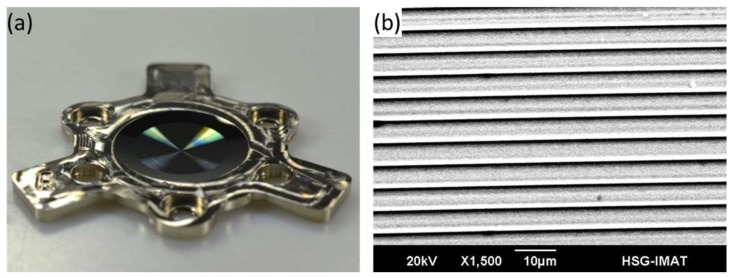
(**a**) Electroplated mold insert with diffractive structure, (**b**) SEM image of the diffractive structure.

For the replication of these kinds of structures, a mold insert has to be fabricated. Therefore, nickel electroplating can be used. The produced structures in a photoresist represent the master which is afterwards cast [130]. The resulting nickel form can then be used as a mold insert for the replication of the structure with the injection molding process. An example of an electroplated mold insert with a diffractive structure is shown in Figure 11.

**Figure 12:**
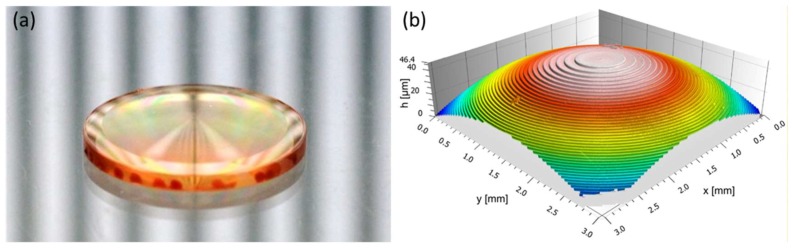
(**a**) Curved glass master with diffractive structure produced by laser direct writing (LDW), (**b**) confocal measurement of the diffractive structure. Reproduced with permission from [133].

Recent developments in LDW made it possible to structure curved surfaces overcoming the limitation of planar substrates [131]. This provides additional freedom for the optical design of specialized optical systems. Structure sizes usually tend to be around 5 µm to obtain sufficient diffraction efficiency of about 70% but can also go down to 1–3 µm with further reduction of the efficiency [132]. The achievable surface roughness is reported to be 25 nm, naming the raster scanning and positioning errors as the main influences [114]. An example of a curved substrate with diffractive micro-structures produced by LDW is shown in Figure 12.

### 4.4. E-Beam Writing

**Figure 13:**
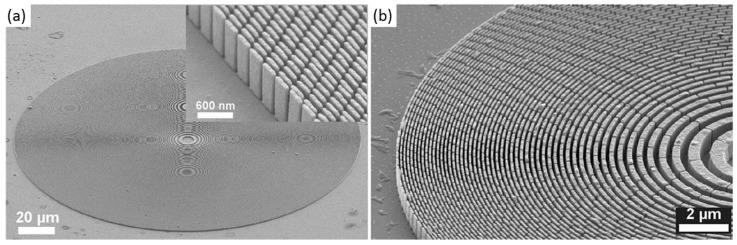
(**a**) Fresnel zone plate fabricated by e-beam writing, (**b**) magnified fiew of the fresnel zone plate. Reproduced with permission from [138].

Electron beam writing is an alternative method for the structuring of a photoresist. Similar to the LDW technology, it is used for the fabrication of a master structure with a subsequent nickel electroplating process. The technology was originally developed for semiconductor mask writing but can also be used for the fabrication of micro optics. E-beam writing is especially suitable for the generation of Fresnel and diffractive structures [134,135]. Figure 13 shows a Fresnel zone plate fabricated by e-beam writing. By scanning the electron beam over a substrate, continuous relief microstructures can also be produced. Thereby the writing area of the electron beam is limited to a few millimeters. For the fabrication of larger substrates, the parts can be moved on a controlled xy-stage. To obtain high-resolution structures, the positioning has to be very accurate. The scanning approach allows a high flexibility for the writing process but results in long writing times.

It is worth mentioning that for the e-beam process additional arrangements are necessary compared to LDW. To avoid charging effects, an additional conducting film under the photoresist is necessary. Furthermore the process has to be performed under vacuum conditions. A detailed description of the electron beam process can be found in [136]. Since the technology is used in semiconductor processes, a lot of effort has been made to push the achievable resolution to the limits. The resolution of e-beam writing in a PMMA-based photoresist can be lower as 10 nm [137]. Similarly to the LDW process, the nickel electroplating process can be used to transfer the structures into a mold insert.

The technology can also be used as a polishing process for metal surfaces. Therefore, a defocused electron beam is used with a spot of some hundred microns and scanned over the surface [139]. Thereby, the metal surface melts which leads to a reduced surface roughness. Using this method a large area can be smoothed within minutes. Uno et al. reported a reduction of the surface roughness from Rz = 6 µm to Rz = 1 µm using this method [140]. Another polishing method using the e-beam technology uses an explosive electron emission applied on a surface without focusing the e-beam. Thereby, the surface roughness can be reduced from Ra = 1 µm to Ra = 0.2 µm [139]. Moreover the e-beam polishing treatment improves corrosion and oxidation resistance significantly [141].

### 4.5. Ion Beam Lithography

Ion beam lithography uses a focused ion beam (FIB) to scan a surface and, thereby, very small structures can be created. The technology is very similar to e-beam writing, but the ions are much heavier and heavier charged. The ion beam has a smaller wavelength than electrons and, therefore, the resolution is even higher than in case of e-beam lithography [142]. Like e-beam lithography, ion beam lithography is a direct writing process. Therefore, there is a lot of freedom in the structuring design but also processing time tends to be long. Structure sizes smaller than 5 nm have been reported using FIB [143]. Early applications for FIB were mainly failure analysis of integrated microelectronics, which is still an important field of activity [144]. Nowadays further applications are the fabrication of micro-channels, micro-structured optics and waveguides [145,146,147]. A subsequent electroplating process is necessary to transfer the micro-structures into a mold insert, mostly provided as sheet metal as a nickel shim.

The technology is also used as a polishing method for lithographic optics. These optics require ultra-smooth surfaces on lenses with 170 mm diameter and larger. Using the FIB method with low energy ions to remove form errors and reduce roughness achieves surface roughness Ra <1 nm [148].

### 4.6. Laser Machining

**Figure 14:**
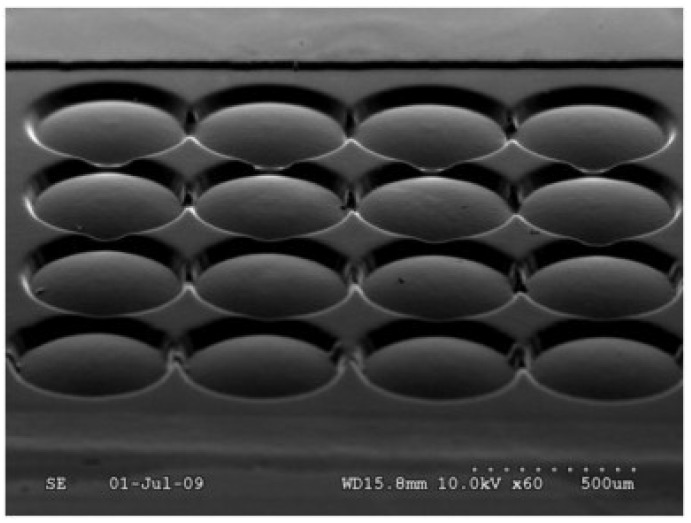
Laser-machined micro lens array with aspheric shape using an excimer laser Reproduced with permission from [155].

The use of short and ultrashort laser pulses is an upcoming technology for different micromachining applications. The method can be used for structuring molding tools [141,149]. Thereby, high-intensity short or ultrashort laser pulses are used for creating micro-features as shown in Figure 14. Pulse width can reach from nanoseconds down to femtoseconds. Examples for applied lasers are excimer, CO_2_ or Nd:YAG lasers. An extensive review of laser beam micro machining is given in [150]. A main advantage of laser machining is the fact that virtually all materials can be machined [151]. The resulting quality very much depends on the combination of laser, workpiece and process parameters. When all these parameters are optimized, laser machining can even be used as a polishing treatment resulting in surface qualities Ra <1 µm [152,153]. Structures with dimensions down to 10 µm can be produced by laser machining [99]. Since the laser spot represents a well-defined energy input, complex structured workpieces can be polished and machined as well. Laser polishing is reviewed by Bordachev et al. in [154].

### 4.7. Polishing/Lapping

Polishing is a finishing treatment with undefined cutting edge to create smooth surfaces with very low roughness. Polishing is not a structuring process, therefore the geometry has to be formed by a different technology in advance. There is a broad range of process variations, but all have in common the fact that an abrasive material is used to smooth a surface. An overview on existing polishing processes is given by Yuan et al. [156]. The abrasives are suspended in a fluid. The suspended abrasive is called slurry. Polishing can create very high surface qualities in the nanometer and sub-nanometer range [157], but the removal rate is typically very low. To create this kind of high quality surface the polishing process, grain size of the abrasive, and polishing time have to be chosen carefully. Polishing can be used to machine plane, spherical, aspherical and freeform workpieces as well as structured surfaces [158,159].

Similar to polishing, lapping uses an abrasive to smooth the surface. The abrasive is rubbed between two surfaces, which can be done by hand or using machines. Lapping is mainly used when high form accuracy is needed. In contrast to polishing, the removal rate is comparatively high. Therefore, the applied grain sizes of the abrasives are usually larger. However, the transition between lapping and polishing is blurry. Both technologies are based on the same material removal mechanism. Since polishing and lapping are mainly used as finishing treatments, previous machining steps have to be performed for the form shaping. Those machining steps are usually done with cutting technologies like (UPM-) turning, milling or variations of these technologies.

An overview of all previously described technologies is shown in Table 1. All technologies are listed with their achievable surface quality and structure size as well as their advantages and limitations.

## 5. Summary and Discussion

The available technologies for the fabrication of optical mold inserts enable the production of high-quality optical parts that can compete with glass optics and replace them in a multitude of applications. To obtain high-quality optical polymer components, deep process knowledge and control during the fabrication of the mold insert as well as during the replication process are necessary. Expanding the know-how in the areas of mold fabrication and polymer replication will open new possibilities for polymer optics and replace glass lenses in further applications.

The challenge for an engineer is to determine the best suitable option for the mold fabrication, which requires a complete understanding of all technologies and fabrication methods and their limitations. This review on different available technologies provides an overview of the technologies as well as a structure to categorize the methods and their capabilities. In the following section, the advantages and disadvantages are summarized in order to support the selection process.

### 5.1. Form-Giving Technologies

The most common technology to produce regular optical mold inserts for spheric and aspheric optical components is ultra-precision machining. It combines high accuracy with optical surface quality in one machining process without necessity of a post-treatment. However, material removal rate is very small, usually down to 1–2 µm for the finishing cut. Therefore, the geometry has to be pre-machined using a regular machining process like turning or milling.

For complex geometries, slow- and fast-tool servo processes as well as diamond milling can be used. Being able to machine complex geometries like non-rotational symmetric parts and free forms is a major advantage of the UPM technology. The fabrication of optical mold inserts by means of ultra-precision machining is time- and cost-consuming since a coating process of the mold insert is necessary. However, improvements in the UPM technology now allow the machining of steel materials, overcoming the limitations of diamond tools. In particular, ultrasonic vibration cutting as well as alternative cutting materials like binderless cBN are powerful technologies to fabricate optical mold inserts in steel-based materials. UPM remains the most promising technology when accurate form-giving is required in an optical mold insert. When complex geometries are required, no other technology offers as much freedom in the design as UPM.

EDM offers the possibility to produce very accurate forms at comparatively high removal rates. However, achievable surface quality is not sufficient for optical applications. Further technological improvements are necessary to enable form-giving fabrication of optical mold inserts without additional post-treatment. EDM is a powerful machining process when mold inserts with high aspect ratios need to be fabricated, however improvements in surface quality are mandatory.

ECM is a relatively new approach as a machining technology for optical mold inserts. The possibility to machine even hardened materials is a main advantage of the technology. Optical surface quality is reported, but a lot of process know-how is necessary to achieve this kind of surface quality. Still, compared to ultra-precision machining, the surface roughness is significantly higher.

Grinding is a form-giving machining technology applied to create very accurate forms. However, the technology is limited concerning the achievable geometry and surface quality. The use of optimized grinding wheels like cBN-wheels as well as in-process dressing methods achieves surface qualities in the sub-micrometer range. Post treatments like polishing or lapping can be used to optimize surface quality when a regular grinding process is used or surface roughness has to be further improved.

### 5.2. Micro-Structuring Technologies

To fabricate micro-structured mold inserts, a broad range of technologies is available. Finding the best method is even more challenging compared to form-giving technologies. Critical factors to consider are the size of the structured area, continuous or binary structures, aspect ratio and substrate material. UPM is not just a suitable method to shape a mold insert but can be also used to create micro-structures. In particular, the fly-cutting process can fabricate large structured areas in the centimeter range in a fast and cost-efficient way, but the achievable geometry is limited. To fabricate rotationally symmetrical structured mold inserts, diamond turning is a perfectly suited method. For all micro-structures created by UPM, the available diamond tools limit the structure geometry as well as the structure size. A big advantage of the UPM technology for the fabrication of micro-structures is the fact that structuring happens directly in the mold insert and no subsequent casting process like electroplating is necessary. This reduces fabrication costs and time as well as possible inaccuracies due to a multitude of process steps.

Similar to UPM, the EDM process can also be used to create micro-structures. EDM is applied for micro structures with several micrometer or tens of micrometer in size and the aspect ratio can be high. The technology can be used to structure steel molds directly, which is a major advantage of the process compared to other micro-structuring methods.

The LIGA process is a well-established technology to produce optical mold inserts with micro features. The fabrication of diffractive gratings has been done by LIGA for a very long time. The technology allows the structuring of areas in the centimeter range but is limited to flat substrates. Part of the LIGA process chain is a galvano-copying step, creating a hard mold insert made out of nickel. Geometries of the micro-structures range from diffractive gratings, micro lenses and micro prisms to waveguides. The application of the LIGA process is advisable when high aspect ratio and accuracy is required and the micro structures are very small (<1 µm). However, the process becomes very complex and expensive, when many hierarchic levels are needed. Then, the lithography process becomes challenging.

NIL is a micro-structuring technology to produce very small structures at the highest possible accuracy. Part of the process is the fabrication of a mold insert. For the fabrication of the mold, micro-structuring technologies like lithography or e-beam writing are used. NIL can be used to produce micro-structured polymer parts, so it is not a typical technology for producing mold inserts. Nevertheless, electroplating can be used to create a mold insert by either casting the master mold or a structured photoresist. Similar to the LIGA process, high aspect ratios are possible. The technology is very well suited for applications where very small (<1 µm) and accurate micro-structures are needed.

LDW is a technology that is very well suited for the fabrication of Fresnel and diffractive structures. A big advantage of the technology is the possibility of creating continuous micro-structures. Especially for diffractive optical elements the efficiency of the elements can be increased significantly. Another advantage of the technology is the possibility to create micro-structures on curved substrates. Since the structure is written into a photoresist, the fabrication of an optical mold insert needs a subsequent electroplating process. The achievable structure size is mainly limited by the spot size of the laser. The process is advisable when feature sizes are larger than 1 µm.

E-beam writing is a micro-structuring technology that can be used when very small micro-structures in the submicron range are required. A major limitation of the process is the processing time, limiting the structured area to the micro-meter/millimeter range. In particular, for the fabrication of small Fresnel or diffractive structures with structure sizes <1 µm, e-beam writing can make use of its technological advantages. Since the process is usually applied to a photoresist, a subsequent electroplating process is necessary.

Laser machining comes with the same obstacle as the LDW process, with the limitation of the structure size to the laser sport size. However, a major advantage of the laser-machining process is the freedom to choose the substrate material. Micro-structures can be directly machined into a mold insert, eliminating a subsequent electroplating process. Since the process is a scanning method, the structuring area is limited to small areas, otherwise processing time increases significantly. Suitable applications for the laser machining process are diffractive gratings and waveguides.

**Figure 15:**
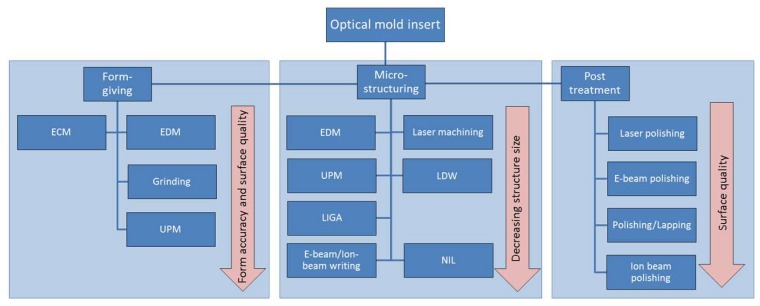
Available technologies for form-giving machining, micro-structuring and post-treatment.

To support the decision on finding a suitable fabrication method, a guide is given in Figure 15. Thereby, three categories are distinguished, namely form-giving, micro-structuring and post-treatment. For each category, the technologies are ordered according to the most important factor of the category. Grinding and UPM enable high accuracy and good surfaces as form-giving methods, however the material removal rate decreases significantly compared to ECM and EDM. For micro-structuring technologies, the achievable structure sizes are an important factor. As a rule of thumb it can be said that with decreasing structure size and increasing form accuracy, the area that can be structured decreases due to long processing time. For all machining methods where the resulting surface quality is not sufficient for optical applications, post-treatments enable a subsequent enhancement of the surface quality. Especially, polishing and lapping enable the fabrication of optical surfaces. However it needs to be considered that the overall form and form accuracy might be affected by a post-treatment process.

As presented in this review, a broad range of technologies is available. Improvements in replication techniques like hot embossing, injection molding, injection compression molding and roll-to-roll replication enable the fabrication of high-performance optical components. Further developments in this area will open new possibilities and applications for polymer optics. In particular, in combination with precise micro-assembly technologies, these optical components can be integrated into optical systems to increase performance and reduce weight.

Increasing requirements of the optical components in accuracy and structure size also needs improvements in the available metrology. The combination of sub-micron resolution with large measurement areas as well as complex geometry remains a challenge for existing measurement systems [160]. Merging high-end mold-fabrication technologies, accurate replication methods and precise micro-assembly methods will enable new applications of polymer optics in optical systems as well as upcoming fields like quantum sensors and plasmon effects.

## Figures and Tables

**Table 1:** Overview of technologies to fabricate optical mold inserts and micro-structured molds.

Method	Surface Roughness	Micro-Structuring	Advantages	Limitations	Ref
Ultra-Precision Machining (UPM)	-	-	-	Available diamond tools limit size and shape of micro-structures	[46]
Diamond Turning	<5 nm	5 µm	Very high accuracy and surface quality	Limited to symmetrical parts and non-ferrous materials	[47]
Slow Tool Servo (STS)	<10 nm	5 µm	Fabrication of asymmetrical parts	Geometries are limited due to the slow stroke of the tool	[54]
Fast Tool Servo (FTS)	<10 nm	<1 µm	Fabrication of asymmetrical parts, fast and accurate positioning of the tool	Geometry has to be within the scope of the FTS stroke	[63]
Diamond Milling	<10 nm	50 µm	Fabrication of free-form structures	Long machining time especially when good surface quality is required	Exp. data
Fly Cutting	<10 nm	<1 µm	Fabrication of complex microstructures like prisms and pyramids	Limited to flat substrates	Exp. data
UPM of steel	<10 nm	5 µm	Machining of ferrous materials with high accuracy	Ultrasonic vibration cutting is limited to a turning process, other methods have problems with wear	[80]
Electric Discharge Machining	<0.1 µm	<10 µm	Large material removal rate	Only conductive workpieces, surface roughness not sufficient for optical applications	[91,96]
Electrochemical Machining	30 nm	Not suitable as a micro-structuring technique	No tool wear, high removal rate also in hardened materials	Only conductive workpieces, electrodes can be complex and expensive	[98]
Grinding	<10 nm	Not suitable as a micro-structuring technique	Machining of hardened steel	Long machining time	[107]
Lithographie, Galvanik and Abformung (LIGA)	<10 nm	<1 µm	Micro-structures with high aspect ratio are possible, broad range of micro-structures is possible	Limited to flat substrates, expensive and complex when multiple lithography steps are necessary	[113]
Nanoimprint Lithography	-	<10 nm	Fabrication and replication of very small micro- and nano-structures, high throughput	Quality is very much depending on the stamp which has to be fabricated by a micro-structuring technology, limited to 2D substrates	[126]
Laser Direct Writing	25 nm	1–3 µm	Suitable for curved substrates, fabrication of continuous structures	Limited to structuring of a photoresist	Exp. data
E-Beam Writing	0.2 µm	<100 nm	Machining of all materials, suitable for large area smoothing	Limited to small areas due to long process time	[139]
Ion Beam Lithography	<1 nm	<10 nm	Machining of all materials except for magnetic materials, fabrication of nano- and micro-structures	Limited to small areas when used as a structuring method due to long process time	[148]
Laser Machining	0.2 µm	10 µm	Processing of every material	Resulting surface quality not sufficient for optical applications	[99]
Polishing/Lapping	<1 nm	Not suitable as a micro-structuring technique	Very high surface quality	Limited form accuracy especially in free-form parts	[156]
